# Altered regulation of negative affect in patients with fibromyalgia: A diary study

**DOI:** 10.1002/ejp.1706

**Published:** 2020-12-09

**Authors:** Silke Rost, Geert Crombez, Stefan Sütterlin, Claus Vögele, Elke Veirman, Dimitri M. L. Van Ryckeghem

**Affiliations:** ^1^ Department of Behavioural and Cognitive Sciences University of Luxembourg Esch‐sur‐Alzette Luxembourg; ^2^ Department of Experimental‐Clinical and Health Psychology Ghent University Belgium; ^3^ Faculty for Health and Welfare Sciences Østfold University College Halden Norway; ^4^ Division of Clinical Neuroscience Oslo University Hospital Oslo Norway; ^5^ Research Group Health Psychology University of Leuven Belgium; ^6^ Department of Clinical Psychological Science Maastricht University Maastricht Netherlands

## Abstract

**Background:**

Fibromyalgia is characterized by widespread musculoskeletal pain and often accompanied by cognitive and emotional problems. Adaptation to fibromyalgia may therefore also rely on one's ability to regulate emotional problems. In this study, we examined two indices of emotion regulation, that is, (a) affective instability, involving frequent large fluctuations in self‐reported affect, and (b) resting heart rate variability (HRV).

**Methods:**

Participants were 46 patients with fibromyalgia (*M*_age_ = 45.4 years; 39 females) and 46 matched healthy controls (*M*_age_ = 44.9 years; 37 females). Heart rate was monitored under resting conditions to derive HRV. Subsequently, participants completed an electronic end‐of‐day diary for 14 consecutive days assessing daily levels of pain, disability, negative affect (NA) and positive affect (PA). Affective instability was operationalized as the mean square of successive differences in daily mood.

**Results:**

Results indicate increased levels of NA instability and reduced levels of HRV in patients with fibromyalgia in comparison with healthy controls. Furthermore, HRV and NA instability were inversely related. Finally, in patients, higher NA instability was related to increased pain disability.

**Conclusions:**

Current findings support the idea that patients with fibromyalgia are confronted with fluctuating emotions. These results may have important implications for treatment as they provide support for the use of emotion regulation skills training in patients with fibromyalgia to impact upon NA instability.

**Significance:**

This study provides novel insight in the link between emotion regulation indices,that is, heart‐rate variability and negative affective (NA) instability, in patients with fibromyalgia, and presents evidence for differences in both emotion regulation indices between patients with fibromyalgia and healthy people. Furthermore, results link increased NA instability with increased levels of daily disability in patients with fibromyalgia. Together, these findings offer support for a key role of emotion regulation in fibromyalgia outcomes, providing pathways for clinical practice.

## 1 INTRODUCTION

Chronic pain patients often experience a myriad of physical, cognitive and emotional problems, challenging daily life tasks (Taylor et al., 2016). Successful adaptation to chronic pain therefore requires the ability to self‐regulate, or exert control over one's bodily symptoms, thoughts, emotions and behaviour (Solberg Nes et al., 2010). Particularly, the regulation of emotions has been argued to be key in the adjustment to chronic pain (Hamilton et al., 2005). Indeed, unsuccessful regulation of high levels of negative affect, often experienced as a consequence of pain, particularly when pain is intense, or its impact (e.g. due to missing days at work or school; Vos et al., 2012) may maintain and/or worsen pain or limit functioning of pain patients (Koechlin et al., 2018). Based on this idea, it has been hypothesized that indicators of impaired emotion regulation, such as increased *affective instability*—that is, the tendency to experience unusually large and/or frequent changes in affect—and decreased levels of *resting heart rate variability* (HRV)—that is, a biomarker of latent emotion regulation capacity (Holzman & Bridgett, 2017; Tracy et al., 2016)—would be associated with worse outcomes in people suffering chronic pain.

For affective instability, initial findings in the field (Rost et al., 2016) indicated that chronic pain patients with increased negative affect (NA) instability showed greater disability. Furthermore, NA instability also moderated the association between daily pain and daily disability ratings, indicating a stronger association between pain severity and disability for patients who are more unstable in their NA. Recently, Gerhart et al. (2018) corroborated these findings by indicating that chronic low back pain patients displaying greater NA variability reported higher average levels of pain and pain interference than their less emotionally variable peers.

For HRV, previous research indicated that chronic pain patients have decreased HRV levels compared to healthy people (Koenig et al., 2016). Yet research has not investigated the role of decreased HRV upon poor health outcomes, such as increased disability levels, in chronic pain patients. This is surprising as the model of neurovisceral integration (Thayer & Lane, 2009) considers HRV as a proxy for prefrontal cortical inhibitory capacity and thus supports behavioural flexibility and adaptive emotion regulation (Appelhans & Luecken, 2006).

Additionally, the link between HRV and daily affective instability is limited to non‐clinical samples (Koval et al., 2013). Although these findings suggest that HRV and affective instability may be complementary indices of emotion regulation, replicating these findings in clinical samples is warranted.

In current study, we investigated whether patients with fibromyalgia differ from matched healthy participants in terms of HRV and day‐to‐day fluctuations in affect. Combining a dynamic measure of daily affective experience and the self‐regulatory biomarker of HRV enables us to expand current knowledge about the role of emotion regulation in fibromyalgia, as well as to explore further links between HRV and affective instability. In particular, we hypothesize that patients with fibromyalgia show higher levels of NA instability, and lower levels of HRV than healthy controls (H1). We furthermore hypothesize that NA instability is negatively associated with HRV in both groups (H2). Additionally, and replicating our previous findings (Rost et al., 2016), we hypothesize that NA instability is related to daily disability (H3a) and moderates the relationship between daily pain severity and disability in patients with fibromyalgia (H3b). For each of the hypotheses, we also explored the role of PA instead of NA instability.

## 2 MATERIALS AND METHODS

### 2.1 Participants

Participants between the ages of 18 and 65 years were recruited as part of a large research project (see ASEF‐I protocol; http://hdl.handle.net/1854/LU‐5686902). Individuals were eligible for participation if they were diagnosed with fibromyalgia (assessed at a multidisciplinary pain clinic) and fulfilled the 2010 diagnostic criteria for fibromyalgia (fibromyalgia group), or if they did not report a current pain problem (control group). Furthermore, participants were excluded if they (a) had insufficient knowledge of the Dutch language; (b) had a neurological condition (e.g. epilepsy); (c) were unable to use both index fingers; (d) reported abnormal sensations on the arm, or (e) did not have normal or corrected‐to‐normal eyesight. The latter three criteria were exclusion criteria related to a task that was not part of the present study (see http://hdl.handle.net/1854/LU‐5686902). Patients with fibromyalgia were recruited via a Multidisciplinary Pain Clinic. They were informed about the study via a poster advertisement in the waiting room of the hospital. Patients who were interested in taking part in this study, left their contact details, and were later contacted to be screened for eligibility. Healthy controls were recruited via advertisements in a local newspaper, flyers and the university website. Individuals who volunteered were contacted and screened for eligibility. Both groups were matched for age, sex and educational level. A total of 98 individuals took part in the study, that is, 49 patients with fibromyalgia and 49 healthy controls.

As the study was part of an extended protocol, we only report the procedure relevant for the current research question (for all study protocol details, see http://hdl.handle.net/1854/LU‐5686902). Before coming to the laboratory, participants completed a questionnaire battery, including the Depression Anxiety Stress Scales (DASS; Lovibond & Lovibond, 1995), Pain Disability Index (PDI; Pollard, 1984) and Multidimensional Pain Inventory (MPI; Kerns et al., 1985; Lousberg et al., 1999) online via LimeSurvey or (for participants who were unable to complete the questionnaires online) with paper and pencil. Upon arrival at the laboratory, the experimenter orally assessed the ACR‐criteria (Wolfe et al., 2010) via the Widespread Pain Index. At the start of the laboratory session, participants’ heart rate was measured for 10 min at rest to derive HRV. At the end of the laboratory session, participants received instructions for a 14‐day diary protocol. The study design was approved by the Ethics Review Panel of the University of Luxembourg and the Medical Ethics Committee from the University Hospital Ghent. Participants gave written informed consent and were reimbursed 35€ for participation.

### 2.2 Questionnaires

*Depressive mood, anxiety* and *stress* were assessed using the DASS (Lovibond & Lovibond, 1995). Each subscale contains 14 items on which participants rate how much they have experienced each state (e.g. ‘I found it hard to wind down’, ‘I felt I was pretty worthless’) over the past week using a scale ranging from 0 (*did not apply to me at all*) to 3 (*applied to me very much, or most of the time*). Scores for each subscale ranged from 0 to 42. In the present study, internal consistencies were excellent (*α* = 0.97 for depression, *α* = 0.91 for anxiety, and *α* = 0.95 for stress).

*Pain severity* at baseline was assessed with the pain severity subscale of the MPI (Kerns et al., 1985; Lousberg et al., 1999). Part I of the MPI consists of five subscales assessing the impact of pain (i.e. pain severity, pain interference, social support, perceived life control and affective distress) on a 7‐point Likert scale. Each subscale has a scale range from 0 to 6. The used pain severity subscale includes two items (i.e. ‘Rate the level of your pain at the present moment’ and ‘On average, how severe has your pain been during the last week’?). A third item (‘How much suffering do you experience because of your pain’?) was not used as its content relates to suffering rather than pain severity (see Parenteau & Haythornthwaite, 2011; Van Ryckeghem et al., 2013). The MPI has shown good reliability and validity (Rudy, 1989). In the present study, Cronbach's alpha of the MPI pain severity subscale was 0.84.

*Pain‐related disability* at baseline was measured with the PDI (Pollard, 1984). In particular, participants indicate the degree of disability experienced in seven life domains (e.g. family and occupation) using a scale from 0 (*no disability*) to 10 (*total disability*). Participants are asked to respond to each category by indicating the overall impact of pain in their life, not just when pain is at its worst. Questionnaire scores range between 0 and 70. In current study, Cronbach's alpha was 0.82.

### 2.3 HRV

To assess HRV under resting conditions, participants were seated in individual cubicles and were instructed to sit quietly and relax while their heart rate was monitored. Heart rate was assessed with electrocardiographic recordings for 10 min at a sampling rate of 1,000 Hz using a Polar watch RS800CX (Polar Electro Oy). HRV was derived from the last 5 min of the recording.

### 2.4 End‐of‐day diary assessment

Participants completed an online diary at the end of each day for two consecutive weeks. They were reminded to do so each evening around 7 p.m. via a text message. The diary took approximately 5 min to complete. Here we describe only the items relevant for the current analyses. Items were developed iteratively by a group of pain researchers and piloted for feasibility in patients with chronic pain.

#### 2.4.1 Daily affect

To assess participants’ daily experience of several affective states, participants answered 16 statements (e.g. ‘Today, I felt enthusiastic’; 0 = *do not agree at all* and 6 = *totally agree*; see also Russell & Carroll, 1999). Particularly, we used six adjectives to measure PA: glad, enthusiastic, happy, relaxed, strong and proud; and 10 adjectives to measure NA: afraid, irritated, angry, powerless, sad, frustrated, dejected, infuriated, hopeless and nervous^1^. Items were derived from a validation study investigating the representation of emotion terms in a general population and a previous study investigating affective instability in chronic pain patients (Rost et al., 2016; Veirman & Fontaine, 2015). PA and NA scales were calculated by averaging PA and NA items respectively. We calculated within‐person reliability of the PA and NA scales using the Generalizability theory approach described by Bolger and Laurenceau (2013). Estimates of within‐person reliability were 0.86 for PA and 0.86 for NA.

#### 2.4.2 Daily pain severity

Daily pain severity was assessed using the item: ‘On average, how severe has your pain been today’? rated on a scale from 0 (*no pain*) to 10 (*worst imaginable pain*).

#### 2.4.3 Daily pain disability

Daily pain disability was assessed by the item ‘To what extent did pain interfere with your planned activities today’? rated on a scale from 0 (*not at all*) to 10 (*very much*). This item is similar to the items in the PDI, but it asks more generally about the degree to which pain prevents patients from their planned daily activities.

### 2.5 Affective instability

Affective instability refers to the experience of frequent and large successive changes in feelings over time (Jahng et al., 2008), and is typically measured using the square of successive differences (SSD; Jahng et al., 2008) which reflects the magnitude of change in consecutively assessed affective states and it is therefore a function of both variability (i.e. average magnitude of affective changes) and temporal dependency (i.e. average frequency of affective changes; Jahng et al., 2008; see further for statistical information).

### 2.6 Data handling and reduction

To calculate HRV‐indices, sequential interbeat intervals were downloaded using the software Polar Pro Trainer 5. All signals were visually inspected for artefacts. HRV analysis was performed using ARTiiFACT (Kaufmann et al., 2011). First, measurement artefacts were identified by applying a distribution‐related threshold criterion. Erroneous beats were deleted and substituted by cubic spline interpolation of neighbouring intervals. Time domain measures were directly calculated from RR‐interval series. Spectral analysis of the RR‐interval series was carried out using Fast Fourier Transformation. Following the recommendations of the Task Force of the European Society of Cardiology and the North American Society of Pacing and Electrophysiology (1996), we defined the high frequency band (HF) as 0.14 to 0.4 Hz and used the following time and frequency HRV parameters for statistical analyses: root mean square of successive differences (RMSSD) and the absolute power in the HF band (HFabs; Task Force of the European Society of Cardiology & the North American Society of Pacing & Electrophysiology, 1996). We focused on those parameters because they closely reflect parasympathetic control over heart rate (Task Force of the European Society of Cardiology & the North American Society of Pacing & Electrophysiology, 1996), which is considered directly relevant to an individual's capacity to regulate emotions (Appelhans & Luecken, 2006; Thayer & Lane, 2000, 2009). The criterion for outliers in HRV measures was defined as values more than 3 *SD* above the sample mean (cf. Koval et al., 2013). After correcting for outliers, HFabs was log transformed to adjust for skewness of the distribution (lnHFabs).

To model affective instability, following Jahng et al. (2008), we conducted analyses using squared successive differences (SSDs), which were calculated by subtracting each participant's reported affect level on a given day from their affect level reported on the following day for each of the 14 days. This resulted in a time series of up to 13 successive (day‐to‐day) differences for each participant, which were squared to obtain SSDs. SSDs were calculated separately for PA and NA ratings. SSDs were log transformed to adjust for skewness of the distribution. Skewness values for the SSD before log‐transformation were 4.6 (PA) and 4.1 (NA), which decreased to −0.78 (PA) and −0.46 (NA) after log transformation. In addition, the MSSD index was calculated for NA and PA, reflecting the average frequency and size of day‐to‐day fluctuations in affect over 14 days (see Houben et al., 2015; Rost et al., 2016).

One control participant was excluded from the final analyses due to equipment failure. A further two participants (1 patient, 1 control) were excluded because they were outliers on HRV. To ensure that affective instability could be modelled reliably, three additional participants (2 patients, 1 control) who completed fewer than 7 out of 14 days of diary entries were excluded from analyses. The final sample consisted of 46 patients with fibromyalgia and 46 healthy controls. For these participants, 93.79% of requested dairy entries were filled out.

### 2.7 Statistical models

Descriptive statistics were calculated using SPSS 24.0 for Windows (SPSS Inc.). For our main analyses we ran multilevel regressions using HLM (Version 7.01; Scientific Software International) to account for the nested structure of the data (daily diary reports nested within individuals) and handle missing data at level 1. All multilevel models included random intercepts and slopes and were estimated using full maximum likelihood.

#### 2.7.1 Model 1: Affective instability as a function of HRV in patients with fibromyalgia and healthy controls

Model 1 was built to test group differences in affective instability and HRV (H1), and negative association between affective instability and HRV in both groups (H2). We modelled the (log transformed) within‐person SSD (lnSSDaffect*_ij_*) using a multilevel random intercept model, in which the Level 1 random intercept (*β*_0_ *_j_*) was predicted by vagally mediated HRV and group (dummy coded 0 = control; 1 = fibromyalgia) at Level 2. We controlled for the mean of daily PA or NA ratings (at Level 2) to ensure that findings were not driven by differences in prevailing affect levels (Ebner‐Priemer et al., 2009; Russell et al., 2007). Vagally mediated HRV and mean affect were standardized to facilitate interpretation (Nezlek, 2012), and group was entered uncentred. PA and NA instability were modelled in separate analyses and the equations, including the subscripts *i* representing days and *j* representing persons, were as follows:

$$\text{Level}\;1:{\text{ln(SSDaffect}}_{\mathrm{ij}})=\beta_{0j}+r_{\mathrm{ij}}$$

$$\text{Level}\;2:\beta_{0j}=\gamma_{00}+\gamma_{01}*{\text{group}}_{j}+\gamma_{02}*{\text{HRV}}_{j}+\gamma_{03}*\text{mean}\;{\text{affect}}_{j}+\mu_{0j},$$

#### 2.7.2 Model 2: The relationship between affective instability and daily disability in patients with fibromyalgia

Model 2 was built to test the positive association between affective instability and daily disability (H3a) and the moderating effect of affective instability on the relationship between daily pain severity and disability in patients (H3b). We modelled daily disability using a multilevel random intercept and slope model. We ran analyses *exclusively in patients with fibromyalgia* as the variance of the diary data of healthy control subjects was limited, that is, ratings were ≤2 on scales from 0 to 10 in 85% for daily pain severity and 90% for daily disability. We followed a model building procedure in our analyses (Raudenbush & Bryk, 2002). To maximize stability and reliability of the findings, we excluded control variables from further steps in model building if their effects proved to be non‐significant (Kreft & de Leeuw, 1998). Level 1 variables consisted of the daily diary measures of pain intensity and pain disability. All level 1 variables were continuous and entered person‐mean centred (Enders & Tofighi, 2007). At level 2, in addition to the main predictors mean levels of daily affect and affective instability, we also included gender, age, baseline disability, baseline pain intensity and pain duration as control variables. Gender was dummy coded (0 = female; 1 = male) and entered uncentred, whereas all continuous Level 2 variables were standardized to facilitate interpretation (Nezlek, 2012). We controlled for mean level of daily affect when investigating the moderating role of affective instability in the final models:

$$\text{Level}\;1:\text{daily}\;\text{disability}=\beta_{0j}+\beta_{1j}*\text{daily}\;\text{pain}\;{\text{severity}}_{\mathrm{ij}}+r_{\mathrm{ij}}$$

$$\begin{array}{cc} \text{Level}\;2 & \beta_{0j}=\gamma_{00}+\gamma_{01}*\text{mean}\;\text{daily}\;{\text{affect}}_{j}+\gamma_{02}*\text{affect}\;{\text{instability}}_{j}+\mu_{0j}\\ & \beta_{1j}=\gamma_{10}+\gamma_{11}*\text{mean}\;\text{daily}\;{\text{affect}}_{j}+\gamma_{12}*\text{affect}\;{\text{instability}}_{j}+\mu_{1j} \end{array}.$$

## 3 RESULTS

### 3.1 Participant characteristics and group differences

Table 1 shows sample demographics and descriptive statistics for all measures. There were no significant group differences in age, sex or educational level (all *p*s > .441). For patients with fibromyalgia, the mean pain duration was 189.9 months (*SD* = 117.8). Mean pain severity was 3.62 as measured by the MPI (*SD* = 1.10) and mean disability was 41.9 on the PDI (*SD* = 10.5). These mean levels are comparable with previous studies of chronic pain patients (*M*_MPI_ = 4.2, *SD*_MPI_ = 1.1, *M*_PDI_ = 44.6, *SD*_PDI_ = 13.4; Chibnall & Tait, 1994; Nicholas et al., 2008). Furthermore, patients with fibromyalgia had significantly higher scores on depression [*t*(72) = 3.98], anxiety [*t*(64) = 6.77], and stress [*t*(90) = 4.53] than healthy controls (all *p*s < .001).

**TABLE 1:** Descriptive statistics by participant group

Variable	Group		Difference test
	Fibromyalgia (*n* = 46)	Control (*n* = 46)	
Age (*M, SD*)	45.4 (9.2)	44.9 (12.2)	*t*(84)^a^ = 0.14, *p* = .885, *d* = 0.05
Sex (*n* women)	39	37	*χ* ^2^(1) = 0.303, *p* = .582, *w* = 0.06
Educational level			*χ* ^2^(2) = 1.64, *p* = .441, *w* = 0.13
College/University	41.3%	54.3%	
Secondary school	54.3%	41.3%	
Primary school	4.3%	4.3%	
DASS‐depression (*M, SD*)	13.0 (10.9)	5.6 (6.4)	*t*(72)^a^ = 3.98, *p* < .001, *d* = 0.83
DASS‐anxiety (*M, SD*)	11.1 (7.5)	2.8 (3.6)	*t*(64)^a^ = 6.77, *p* < .001, *d* = 1.39
DASS‐stress (*M, SD*)	15.0 (7.6)	7.9 (7.4)	*t*(90) = 4.53, *p* < .001, *d* = 0.95
Mean daily PA (*M, SD*)	2.6 (1.0)	3.4 (1.0)	*t*(90) = 3.75, *p* < .001, *d* = 0.81
Mean daily NA (*M, SD*)	1.4 (0.7)	1.2 (0.9)	*t*(90) = 1.28, *p* = .204, *d* = 0.29
Ln NA instability (*M, SD*)	−1.6 (1.1)	−2.5 (1.5)	*t*(90) = 3.29, *p* = .002, *d* = 0.69
Ln PA instability (*M, SD*)	−1.3 (1.1)	−1.7 (1.1)	*t*(90) = 1.43, *p* = .157, *d* = 0.30
HRV‐indices			
RMSSD (*M, SD*)	19.1 (10.9)	26.4 (14.0)	*t*(90) = 2.77, *p* = .007, *d* = 0.58
lnHFabs (*M, SD*)	4.7 (1.3)	5.2 (1.2)	*t*(90) = 1.92, *p* = .058, *d* = 0.40

Abbreviations: DASS, Depression, Anxiety and Stress Scales; HRV, heart rate variability; lnHFabs, log transformed absolute power in the HF band; NA, negative affect; PA, positive affect; RMSSD, root mean square of successive differences.

Furthermore, relative to the control group, patients with fibromyalgia showed lower mean levels of daily PA (*t*(90) = 1.28, *p* < .001), but did not differ significantly in their mean levels of NA (*p* = .204). Table 1 gives an overview of all measures.

### 3.2 Multilevel analyses

#### 3.2.1 Affect instability as a function of HRV in patients with fibromyalgia and healthy controls

**FIGURE 1:**
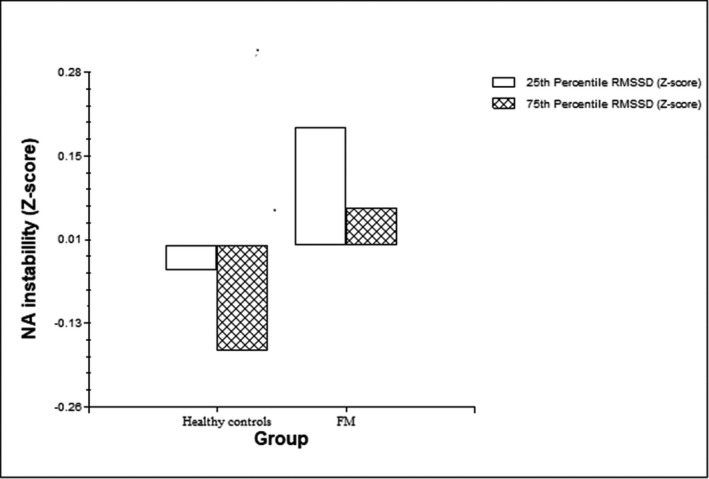
Standardized NA instability scores per Group (healthy controls; fibromyalgia [FM] patients per Standardized HRV‐RMSSD score (low [25th percentile]; high [75th percentile])

Model 1 tested H1 and H2, that is, patients show higher levels of affect instability and lower levels of HRV and a negative association between affect instability and HRV in both groups. We examined how Group (fibromyalgia vs. control) and each index of HRV were related to NA instability, while controlling for the mean level of NA (Model 1a and 1b; see also Figure 1). As predicted, Group was positively associated with NA instability, indicating that patients with fibromyalgia showed significantly higher levels of NA instability. Furthermore, results indicated that NA instability was negatively associated with HRV‐RMSSD, linking lower vagally mediated HRV to higher NA instability (see Model 1a in Table 2). Results investigating the link between NA instability and lnHFabs pointed in the same direction but failed to reach statistical significance (see Model 1b in Table 2). Results for PA instability showed that PA instability was not related to Group and HRV‐indices, while controlling for the mean level of PA (See Models 1c and 1d in Table 2).

**TABLE 2:** Results of multilevel models predicting instability of positive and negative affect from group and HRV‐indices

Predictor		*B*	*SE*	*P*
Model 1 for NA instability				
Model 1a	Intercept (γ_00_)	−2.32	0.20	<.001
	Group (γ_01_)	0.62	0.26	.021
	Mean daily NA (γ_02_)	0.65	0.14	<.001
	HRV‐RMSSD (γ_03_)	−0.26	0.12	.034
Model 1b	Intercept (γ_00_)	−2.37	0.20	<.001
	Group (γ_01_)	0.71	0.25	.006
	Mean daily NA (γ_02_)	0.65	0.15	<.001
	HRV‐lnHFabs (γ_03_)	−0.15	0.12	.214
Model 1 for PA instability				
Model 1c	Intercept (γ_00_)	−1.49	0.40	<.001
	Group (γ_01_)	−0.02	0.25	.946
	Mean daily PA (γ_02_)	−0.33	0.11	.005
	HRV‐RMSSD (γ_03_)	−0.16	0.11	.160
Model 1d	Intercept (γ_00_)	−1.54	0.16	<.001
	Group (γ_01_)	0.05	0.24	.826
	Mean daily PA (γ_02_)	−0.32	0.11	.006
	HRV‐lnHFabs (γ_03_)	−0.16	0.11	.167

Abbreviations: HRV, heart rate variability; lnHFabs, log transformed absolute power in the HF band; NA, negative affect; PA, positive affect; RMSSD, root mean square of successive differences.

H1 is further supported by significant group differences (*t*‐tests) reporting higher levels of NA instability (*p* < .01) but not of PA instability (*p* = .16) in patients with fibromyalgia compared to healthy controls (see Table 1). Moreover, *t*‐tests comparing groups on HRV supports H1 with regard to group differences in HRV, more precisely lower HRV in patients with fibromyalgia than in healthy subjects (Table 1). Both these results support group differences of NA instability and HRV as hypothesized in H1.

### 3.3 Daily disability and affective instability in patients with fibromyalgia

Model 2 tested H3a and H3b, that is, that affective instability is related to daily disability and moderates the relationship between daily pain severity and disability. This model was tested in the patients’ sample only (see also section ‘statistical models’).

#### NA instability

Based on the model building procedure, we found Age to be a significant predictor for the intercept of Daily disability (*B* = 0.48, *t*(38) = 2.57, *p* = .01), indicating that older patients reported higher levels of daily disability. Therefore, we included Age as a Level 2 predictor in the final model. Final analyses indicated several Level 2 variables as significantly predicting the Level 1 intercept: Age (*B* = 0.49, *t*(42) = 2.20, *p* = .03), Mean level of daily NA (*B* = 0.53, *t*(42) = 2.09, *p* = .04) and NA instability (*B* = 0.41, *t*(42) = 2.03, *p* < .05). These findings indicate that daily disability increased with age, higher mean levels of daily NA and higher levels of NA instability, thus supporting H3a. The slope (i.e. moderating effect of NA instability) was found to be not significant (*B* = 0.06, *t*(43) = 1.27, *p* = .21), thus not supporting H3b.

### 3.4 PA instability

Analyses of the model building procedure revealed that Age (*B* = 0.42, *t*(38) = 2.15, *p* = .038) and Baseline pain severity (*B* = 0.58, *t*(38) = 2.01, *p* = .052) were predictors for the intercept, indicating that older patients and patients reporting greater Baseline pain severity reported more disability in daily life. Both predictors were, therefore, included in the final model. Final analyses showed that Age (*B* = 0.44, *t*(41) = 2.23, *p* = .031) and Baseline pain severity (*B* = 0.68, *t*(41) = 3.27, *p* < .01) remained significant predictors for Daily disability. Also, Daily pain severity (*B* = 0.88, *t*(43) = 13.24, *p* < .001) was a significant predictor of Daily disability in the final Model. Of particular interest, PA instability was not related to daily disability. Also, the slope (i.e. moderating effect of PA instability) was not significant (*B* = 0.03, *t*(43) = 0.63, *p* = .53). Results of the final models predicting daily disability are presented in Table 3.

**TABLE 3:** Final hierarchical linear models iwith regard to daily disability in fibromyalgia patients

Model 2 for daily disability	*B*	*SE*	*p*
Final model for NA			
Intercept (γ_00_)	4.75	0.23	<.001
Mean daily NA (γ_01_)	0.53	0.25	.043
NA instability (γ_02_)	0.41	0.20	.048
Age (γ_03)_	0.49	0.22	.033
Daily pain severity (γ_10_)	0.91	0.06	<.001
Daily pain severity × mean daily NA (γ_11_)	−0.01	0.06	.888
Daily pain severity × NA instability (γ_12_)	0.06	0.04	.211
Final model for PA			
Intercept (γ_00_)	4.78	0.17	<.001
Mean daily PA (γ_01_)	−0.25	0.25	.338
PA instability (γ_02_)	0.01	0.15	.941
Age (γ_03)_	0.44	0.20	.031
Baseline pain severity (γ_04)_	0.68	0.21	.002
Daily pain severity (γ_10_)	0.88	0.07	<.001
Daily pain severity × mean daily PA (γ_11_)	−0.06	0.06	.325
Daily pain severity × PA instability (γ_12_)	0.03	0.05	.533

Abbreviations: NA, negative affect; PA, positive affect.

## 4 DISCUSSION

Within the current study, we investigated group differences between patients with fibromyalgia and healthy controls in terms of affective instability and resting HRV (two indices of emotion regulation capacity). Additionally, the relationship between HRV and affective instability across both groups was examined as well as the relationship between affective instability and daily disability in patients with fibromyalgia. Results of our study can be readily summarized. First, patients with fibromyalgia showed higher levels of NA instability and lower levels of resting HRV than healthy controls. Second, HRV (RMSSD) was negatively associated with NA instability, although not with PA instability. Third, NA instability predicted daily disability. However, in contrast with previous findings (Rost et al., 2016), affective instability did not moderate the association between daily pain severity and pain disability. Each of these findings deserves further exploration.

Within the current study, we observed higher levels of NA instability in patients with fibromyalgia compared to healthy people. This is in line with previous research indicating that chronic low back pain patients display greater NA variability than their spouses do (Gerhart et al., 2018) and similar research comparing levels of NA instability between healthy people and people suffering from other psychological disorders (e.g. Thompson et al., 2012). These findings indicate that experiencing chronic pain is related to increased NA instability. This was observed even when controlling for mean negative emotion levels, showing that NA instability has unique features compared to mean negative emotion levels. These findings may be explained by the fact that chronic pain presents constant challenges to a person, needing adequate coping strategies. Due to these constant challenges, it is likely that in some instances, flexible adaptation of coping strategies to the context fails or that after continuous challenges to cope with chronic pain and/or related problems, people fail to remain coping with these challenges, resulting in a wide variability in negative emotions (Solberg Nes et al., 2009). Besides, differences in variability in negative emotions between healthy people and patients with fibromyalgia, current findings also indicate that both, higher mean levels of negative affect and higher variability in negative emotions, were related to higher daily disability. Current findings replicate our previous finding that mean daily NA and NA instability are significantly related to daily disability in a different, that is, homogenous fibromyalgia, patient sample, suggesting that patients reporting more NA and NA instability experience more disability in daily life (Rost et al., 2016). Furthermore, this is in line with recent findings of Gerhart et al. (2018) indicating that moment‐to‐moment variability of NA is related to daily disability in chronic low back pain patients. These findings provide further support for the suggestion of Hamilton et al. (2005) who proposed that individual differences related to emotional processing and specifically emotion regulation might be an important factor in the adaptation to chronic pain. Koechlin et al. (2018) suggest that this link may be due to the fact that unsuccessful regulation of high levels of negative affect maintains/worsens pain or limits functioning of pain patients. This may on its turn fuel one's level of affective instability again, and as such become a vicious reinforcing circle. Yet, it may also be that persistent pain and emotion dysregulation share similar underlying mechanisms (Linton, 2013). For example, negative repetitive thinking, might operate as a transdiagnostic factor, that is, serve as a driver for emotional and pain‐related problems (Flink et al., 2013; Linton, 2013). Negative repetitive thinking might function in order to downregulate NA in stressful situations such as experiencing persistent pain or emotional distress (Flink et al., 2013). When this form of repetitive thinking occurs in other contexts and spins out of control, however, it becomes a form of ineffective problem solving that drives the development of emotional and physical problems (Eccleston & Crombez, 2007; Linton, 2013).

The assumption that affective instability reflects dysregulated emotion is further corroborated by the finding that HRV and NA instability are associated with each other. HRV is driven by a flexible network of neural structures, which is dynamically organized and allows for behavioural adaptability, thus, indexing regulated emotional responding (Appelhans & Luecken, 2006). The link between vagally mediated HRV has previously been reported as predicting lower levels of emotional instability by Koval et al. (2013) in a healthy sample. Yet, the current study is the first to show a relationship between emotional instability, in particular NA instability, and vagally mediated HRV in a chronic pain population. This link was, however, specific for NA instability, and not for PA instability. This finding is not unexpected and may relate to a lower PA/NA balance in patients with fibromyalgia than in healthy individuals. It could be argued that in patients with fibromyalgia, HRV is mainly associated with the downregulation of NA.

Next, it should be noted that we could not completely replicate our previous findings (Rost et al., 2016), showing that NA instability is related to daily disability, and moderates the relationship between daily pain severity and disability in patients with fibromyalgia (H3b). Indeed, although we found again evidence for a relationship between NA instability and daily disability, we were unable to find support for the moderating effect of NA instability on the relationship between daily pain severity and disability in patients with fibromyalgia. This is in contrast with our previous research showing that the association between daily pain severity and daily disability as well as cognitive complaints is stronger in patients who report higher NA instability compared to patients experiencing lower day‐to‐day fluctuations of NA (Rost et al., 2016). One explanation for the divergence of the current findings from our previous results might relate to insufficient power of the multilevel moderation analysis. The average Level 1 and Level 2 sample sizes are of major importance in determining the detection of cross‐level interactions (Mathieu et al., 2012) and may have been too small to allow for the detection of cross‐level interactions.

Finally, it is worth noting that the current study clearly found differential findings for PA instability and NA instability. This is not surprising as a wealth of research has indicated that PA is relatively independent from NA (e.g. Watson & Clark, 1992). These differential findings were also found in previous research in psychopathology investigating affective instability in natural contexts (Houben et al., 2015; Thompson et al., 2012; Trull et al., 2008). For example, depressed patients show higher NA instability, but not PA instability, compared with healthy volunteers. (Thompson et al., 2012). Similarly, previous research of our group (Rost et al., 2016) only found a link between NA instability and poor pain outcomes. These findings may be explained by the fact that there are fewer fluctuations in PA than NA. Inspection of the SDs of both NA instability and PA instability offers some support for this explanation. Yet, it may also be that our measure of NA instability captures a unique aspect of emotion regulation which is not captured by our measure of PA instability. Future research is warranted on the differential effects between NA and PA instability.

Our findings may have clinical implications as they indicate that therapeutic treatment for chronic pain populations could aim to provide chronic pain patients with strategies to improve regulating negative emotions which are often inherent to the presence of chronic pain. For instance, the inclusion of specific emotion regulation skills training (e.g. Berking & Whitley, 2014) and mindfulness‐based approaches (Grossman et al., 2007; Rosenzweig et al., 2010) may offer promising approaches for the psychological treatment of fibromyalgia.

This study has also several considerations. First, we assessed day‐to‐day fluctuations in affect using end‐of‐day diaries. This is in line with our previous research (Rost et al., 2016). Yet, generalization of current findings to within‐day emotional fluctuations warrants further research. Second, affective instability is a global index that captures temporal variability of emotions (in this case over 14 days). We cannot draw conclusions, however, regarding the individual or contextual factors underlying individual differences in affective instability in our sample. In addition, the amount of emotion terms for PA and NA was imbalanced. Future research may want to balance the number of PA and NA items to avoid potential bias in one or the other direction. Third, the current study investigated the relationship between HRV, emotional instability and disability due to pain. Future research should investigate the link with other psychological variables, such as worry or rumination, to become closer to identifying possible shared underlying mechanism of both emotional and pain‐related problems. Fourth, it should be noted that HRV is not uniquely linked to emotion regulation and as many physiological indicators, it may be influenced by effects of the situation and person–situation interaction. Although resting HRV assessment was highly standardized in the current study, future research may aim to include at least two measurements to aggregate in order to diminish the potential influence of state variables (Bertsch et al., 2012). Fifth, two HRV‐indices were included in the current study. It should be noted that although pointing in the same direction, findings with both indices did not mirror each other exactly. This finding is in line with earlier research, whereby the RMSSD index of HRV has been preferred because of its robust statistical properties (Task Force of the European Society of Cardiology & the North American Society of Pacing & Electrophysiology, 1996). Sixth, one should be cautious to infer causal relationships from our data. Indeed, we did not experimentally manipulate variables, so we cannot exclude that other, hidden and unmeasured variables play a role. Furthermore, the shared neurophysiological mechanisms of emotion regulation, pain perception and physiological correlates such as cortico‐cardiac interaction as expressed in HRV, challenge any attempt of disentanglement of causal pathways and require future research under controlled experimental conditions. Future research addressing the causal nature between HRV and affecting instability and poor health outcomes is therefore warranted.

## DISCLOSURES

There are no conflicts of interest that may arise as a result of this research. This research was supported in part by the grant ‘Self‐Regulation and Pain’ [SEREPA; University of Luxembourg] to Claus Vögele.

## AUTHORS' CONTRIBUTIONS

Silke Rost: Conceptualization, Methodology, Data curation, Formal analysis, Writing ‐ Original Draft; Geert Crombez: Conceptualization, Methodology, Writing ‐ Review & Editing; Claus Vögele: Conceptualization, Methodology, Writing ‐ Review & Editing; Stefan Sütterlin: Writing ‐ Review & Editing; Elke Veirman: Writing ‐ Review & Editing; Dimitri M.L. Van Ryckeghem: Conceptualization, Methodology, Data collection, Writing ‐ Review & Editing.
